# Localized Boron Sites in Large Pore Borosilicate Zeolite EMM-59 Determined by Electron Crystallography

**DOI:** 10.1021/jacs.4c14478

**Published:** 2024-12-09

**Authors:** Jung Cho, Elina Kapaca, Bin Wang, Ross Mabon, Hilda Vroman, Xiaodong Zou, Allen W. Burton, Tom Willhammar

**Affiliations:** † Department of Materials and Environmental Chemistry, Stockholm University, SE-106 91 Stockholm, Sweden; ‡ Corporate Strategic Research, ExxonMobil Research & Engineering Co. Inc., 1545 Route 22 East, Annandale, New Jersey 08801, United States

## Abstract

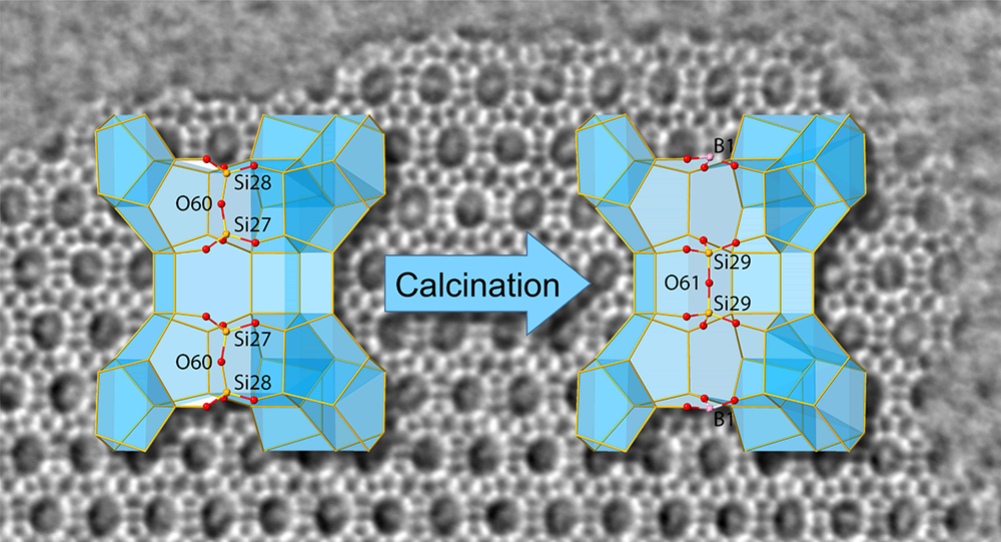

The structure of novel large pore borosilicate zeolite EMM-59 (|C_19_H_42_N_2_|_8_[B_5.2_Si_218.8_O_448_]) with localized framework boron sites was determined by using three-dimensional electron diffraction (3D ED) and scanning transmission electron microscopy (STEM) imaging. EMM-59 was synthesized using 2,2-(cyclopentane-1,1-diyl)bis(*N*,*N*-diethyl-*N*-methylethan-1-aminium) as an organic structure-directing agent (OSDA). The framework has a three-dimensional intersecting channel system delimited by 12 × 10 × 10-ring openings and contains 28 T and 60 oxygen atoms in the asymmetric unit, making it the most complex monoclinic zeolite. The 3D ED data collected from as-made EMM-59 under cryogenic conditions revealed three symmetry-independent locations of the OSDAs, and STEM imaging showed that the OSDAs are flexible and adopt different molecular conformations in channels with identical structural environments. The framework boron atoms were exclusively found in T-sites of 4-rings, especially those shared by multiple 4-rings. The tetrahedral BO_4_ with the highest boron content (38.6%) was transformed into a trigonal BO_3_ after the OSDAs were removed upon calcination. Its location and boron content could also be identified by STEM imaging.

## Introduction

Zeolites are crystalline nanoporous materials extensively used in catalysis and sorption due to their well-defined pore architectures that allow molecules to diffuse through and interact with the bulk of the crystal.^1^ Different arrangements of the tetrahedral TO_4_ (T: Si, Al, B, etc.) building units result in zeolite frameworks with different pore sizes and channel dimensions, enabling shape-selective catalytic properties. To date, there are 256 framework types reported in the IZA Database of Zeolite Structures.^2^ When a silicon atom is substituted by a trivalent heteroatom, typically aluminum, a local negative charge is introduced that must be compensated for by a cation. If it is a proton, the zeolite becomes a Brønsted acid catalyst.^3^

Locating acid sites is important for understanding their local environment and their accessibility by adsorbents for fine-tuning the catalytic properties. However, because of the similar scattering factors of aluminum and silicon, it is difficult to distinguish them by diffraction methods regardless of the radiation source. Instead, acid sites have been located indirectly, such as by using probe molecules.^4−8^ In a recent study, aluminum sites were identified and quantified by exploiting the anomalous scattering at the Al absorption edge by using anomalous powder X-ray diffraction, an experiment that requires a specialized experimental setup and a synchrotron source.^9^ On the other hand, boron, another commonly used trivalent heteroatom, has a substantially different scatting factor from silicon (Figure S1).^10,11^ Therefore, the framework boron atoms can be located and quantified, which can be used to gain structural insights into their role in catalysis and in producing zeolite framework structures inaccessible to aluminosilicate compositions.^12,13^ Furthermore, since large and extra-large pore borosilicates can be converted into aluminosilicates while preserving their native T-sites,^14,15^ the synthesis of borosilicates paves the way for the synthesis of large and extra-large pore aluminosilicates with known locations of Brønsted acid sites. This knowledge facilitates understanding of structure–property relationships and subsequently enables the synthesis of tailored zeolites with desired properties.

An important synthetic parameter that influences the location and distribution of acid sites is organic structure-directing agents (OSDAs). They are typically quaternary ammonium cations used in the synthesis to direct the formation of specific zeolite framework structures and stabilize the negatively charged tetrahedral units with a trivalent T atom.^16^ Since these OSDAs are typically found in the vicinity of T-sites occupied by trivalent heteroatoms,^17,18^ using different OSDAs under identical conditions yields the same framework type but with different locations and distribution of acid sites.^19,20^ OSDAs are confined in the periodic pores of zeolites, and can be located using diffraction techniques.^17,21−23^ This structural information can provide insights into their role in the preferred localization of trivalent heteroatoms and the formation of the framework. However, because OSDAs are typically ammoniums that cannot engage in strong interactions with the framework, they, especially those functionalized with hydrocarbon chains, can assume various molecular conformations within the zeolite pores. Since diffraction data provide only an average structure over the entire crystal, using diffraction alone to determine the atomic coordinates of the OSDAs is often challenging.

Imaging techniques, such as high-resolution transmission electron microscopy (HRTEM) and scanning transmission electron microscopy (STEM), are used to capture local structural information, including faults and disorder,^24,25^ and to determine the structure of low-dimensional zeolitic materials.^26,27^ In particular, the image contrast obtained using (high-angle) annular dark field (HA)ADF-STEM imaging is sensitive to the atomic number, allowing atoms with substantially different atomic numbers to be distinguished. For instance, this technique has been used to study the mechanism of titanium incorporation into the silicate zeolite framework.^28^ Integrated differential phase contrast (iDPC)-STEM imaging is another STEM imaging technique that can enhance the contrast of light elements. This technique was used to reveal adsorbed organic species,^29^ including OSDAs,^30^ and their interaction with the zeolite framework, which helped rationalize the flexible nature of the framework of the industrially important zeolite ZSM-5.^31^ These capabilities make STEM imaging a complementary technique to diffraction for locating framework borons and OSDAs in borosilicates.

Herein, we report the structure of a novel borosilicate EMM-59 that was studied using three-dimensional electron diffraction (3D ED) and STEM imaging techniques. EMM-59 is synthesized with a diquaternary ammonium cation as OSDA with two *N*,*N*-diethyl-*N*-methylamine head groups linked by methylene chains to a central cyclic alkane. The general location of the OSDAs within the pores of as-made EMM-59 is determined using 3D ED, and iDPC-STEM imaging was used to show various molecular conformations of the OSDAs in channels with the same local environment. Furthermore, 3D ED is used to identify T-sites with framework boron atoms in calcined EMM-59 and to determine structural changes at the T-site with the highest boron content after the calcination of the OSDAs, which we further corroborate using ADF-STEM imaging. Based on the determined locations of both the OSDAs and the T-sites with framework boron, we deduce the interaction between the OSDA and the boron-rich T-site.

## Results and Discussion

### Synthesis of EMM-59

**Figure 1:**
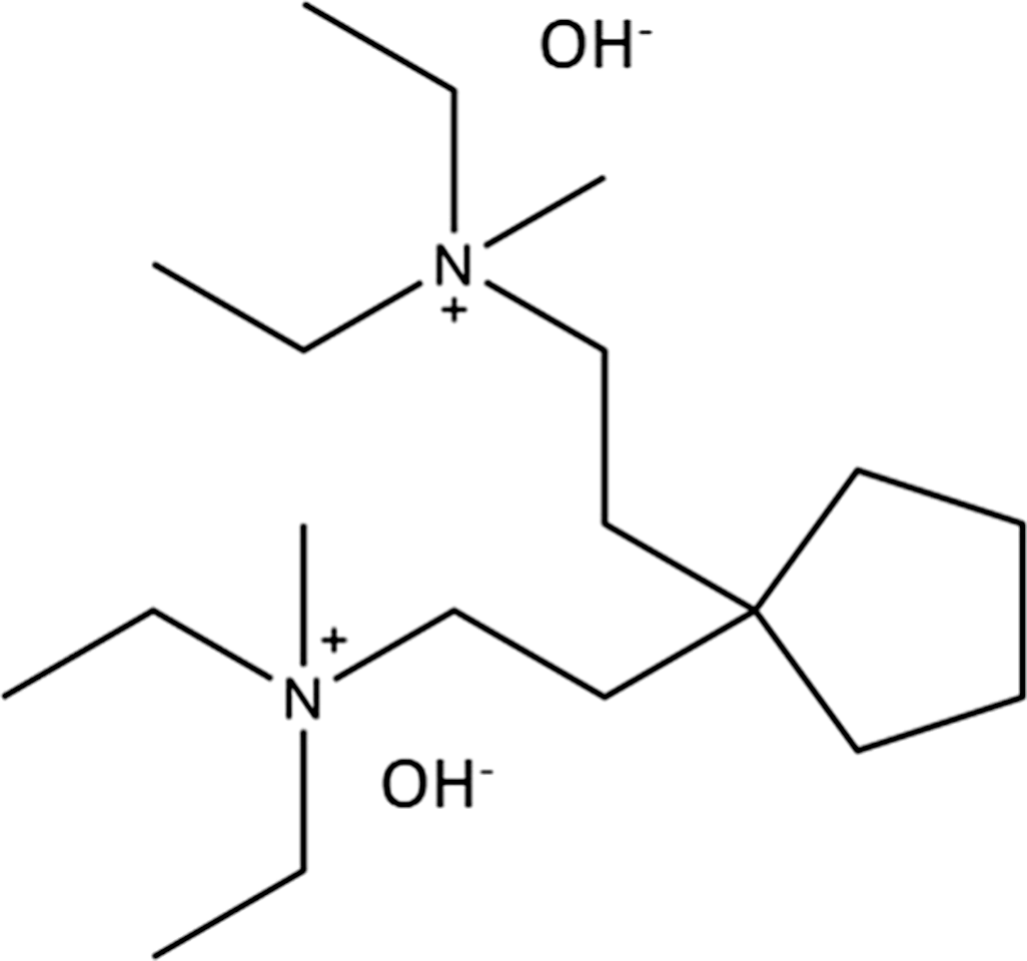
Schematic of the OSDA used to synthesize EMM-59, 2,2-(cyclopentane-1,1-diyl)bis(*N*,*N*-diethyl-*N*-methylethan-1-aminium) hydroxide.

Synthesis of EMM-59 was performed hydrothermally using 2,2-(cyclopentane-1,1-diyl)bis(*N*,*N*-diethyl-*N*-methylethan-1-aminium) as an OSDA (Figure 1). It is worth noting that EMM-59 was only crystallized in the presence of boron. EMM-59 was synthesized from a gel with the following ratios: OH^–^/Si = 0.3, Si/B = 20, K/Si = 0.1, and H_2_O/Si = 30 using KOH together with OSDA–OH (further details can be found in the experimental section). After 14 days, the product was formed with its characteristic powder X-ray diffraction (PXRD) pattern; see Figure S2. The product from this synthesis crystallized as submicrometer-sized flakes (Figure S3).

### Structure Determination and Refinement of As-Made EMM-59

**Figure 2:**
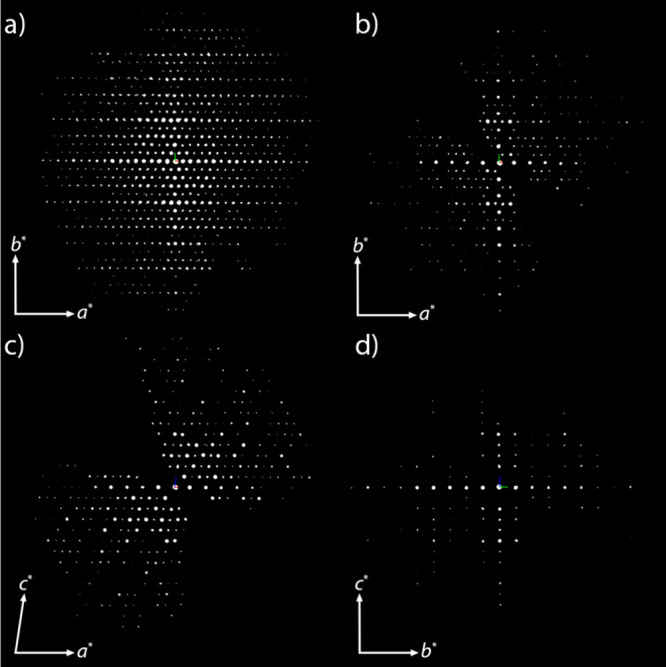
(a) 3D reciprocal lattice of EMM-59 reconstructed from a 3D ED data set. (b–d) 2D slices of the 3D data showing the (b) *hk*0, (c) *h*0*l*, and (d) 0*kl* families of reflections.

3D ED data of the as-made EMM-59 was collected under cryogenic conditions using a cumulative electron fluence of 0.5 e^–^/Å^2^. The 3D ED data could be indexed using a monoclinic unit cell with *a* = 42.44(18), *b* = 19.90(7), *c* = 15.40(9) Å, and β = 97.98(45)°. The reflection conditions can be deduced as *hkl*, 0*kl*, *hk*0: *h*+*k* = 2*n*, *h*0*l*, *h*00, 00*l*: *h* = 2*n*, and 0*k*0: *k* = 2*n* from the 3D reciprocal lattice reconstructed based on the 3D ED data (Figure 2), corresponding to possible space groups *C*2 (5), *Cm* (8), and *C*2/*m* (12). The initial structure obtained in space group *C*2/*m* from SHELXT resulted in a fully tetrahedral structure with 28 Si and 60 O atoms in the asymmetric unit. The least-squares refinement against the 3D ED data converged with an R_1_ value of 28.08% (see Tables S1–3 for details). Locations of boron atoms were not determined from the as-made EMM-59, because of the difficulty in resolving individual atoms of the OSDAs in the pore (*vide infra*).

**Figure 3:**
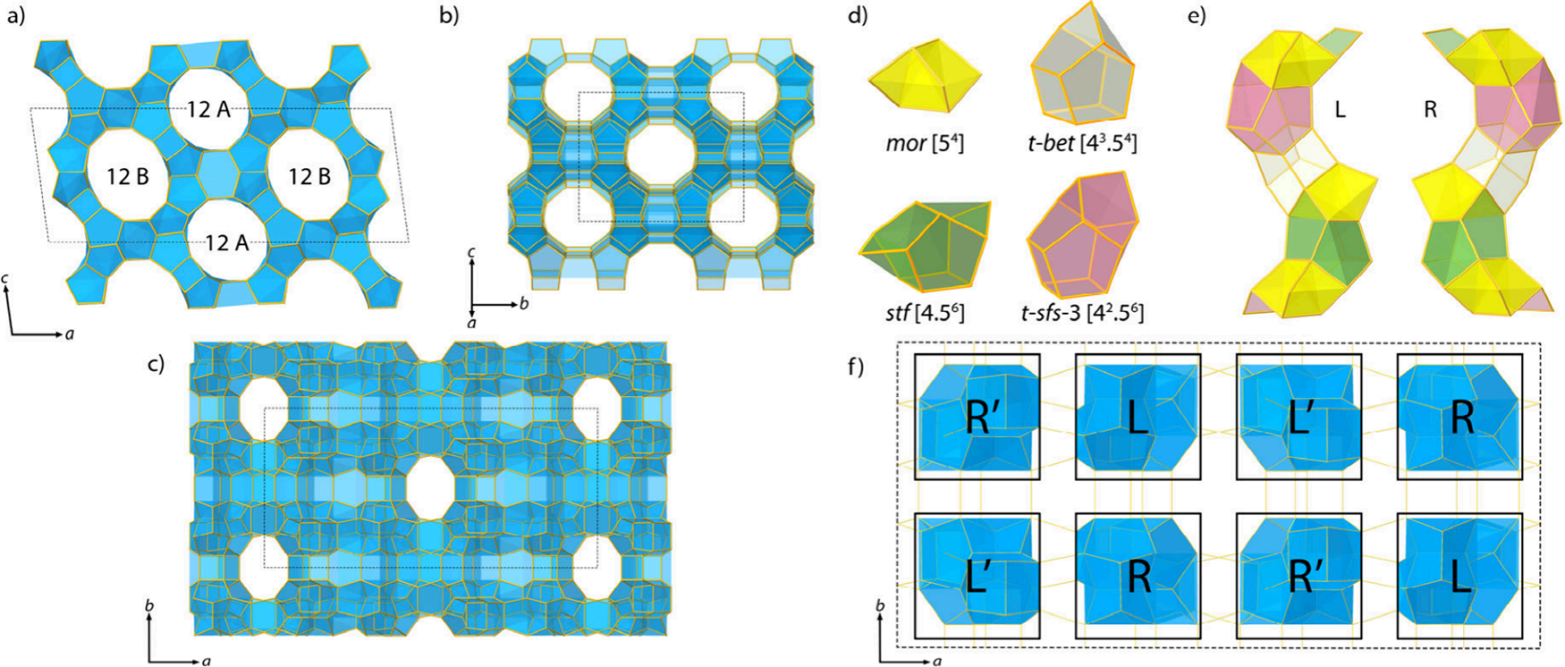
Framework of EMM-59 viewed along the (a) [010], (b) [101], and (c) [001] directions. (a) The two symmetry-independent 12-ring channels (marked 12A and 12B) along the *b*-axis intersect with 10-ring channels along [101] (b) and [001] (c) directions. (d) The four composite building units in the structure of EMM-59 and (e) their connections into left or right-handed columns, denoted L and R respectively. (f) Each unit cell contains four L and four R columns oriented along the [001] direction. The L′ and R′ columns are related to the L and R columns, respectively, by a 2-fold rotation about the *b*-axis.

EMM-59 possesses a three-dimensional channel system of intersecting channels with 12 × 10 × 10-ring openings. The structure consists of two distinct straight 12-ring channels extending along the [010] direction with openings of 7.2 × 5.9 Å (12A) and 8.4 × 6.3 Å (12B) (Figure 3a). The two channels are woven together by a 10-ring channel with an opening of 5.3 × 5.6 Å along the [101] direction (Figure 3b) and a 10-ring channel with an opening of 5.5 and 5.4 Å along the [001] direction (Figure 3c). In the intersections between 12- and 10-ring channels, there are two different types of cavities. Among the twenty-eight framework types with borosilicate composition reported in the IZA Database of Zeolite Structures, seven have a three-dimensional channel system, of which only four have large pore framework types.

The EMM-59 framework is built from four composite building units of zeolites *mor*, *stf*, *t-bet* and *t-sfs-3* (Figure 3d), which are connected to form columns extending along the *c*-axis. The column can adopt two enantiomeric forms, denoted as L and R (Figure 3e). There are 8 columns in each unit cell, arranged into a 4 × 2 matrix according to the sequence [R′LL′R/L′RR′L] (Figures 3f and S5). The L′ and R′ columns are related to L and R columns, respectively, by a twofold rotation about the *b*-axis. Similar arrangements of columns are found in the structures of zeolites ZSM-5 and ZSM-11 (Figure S4).

**Figure 4:**
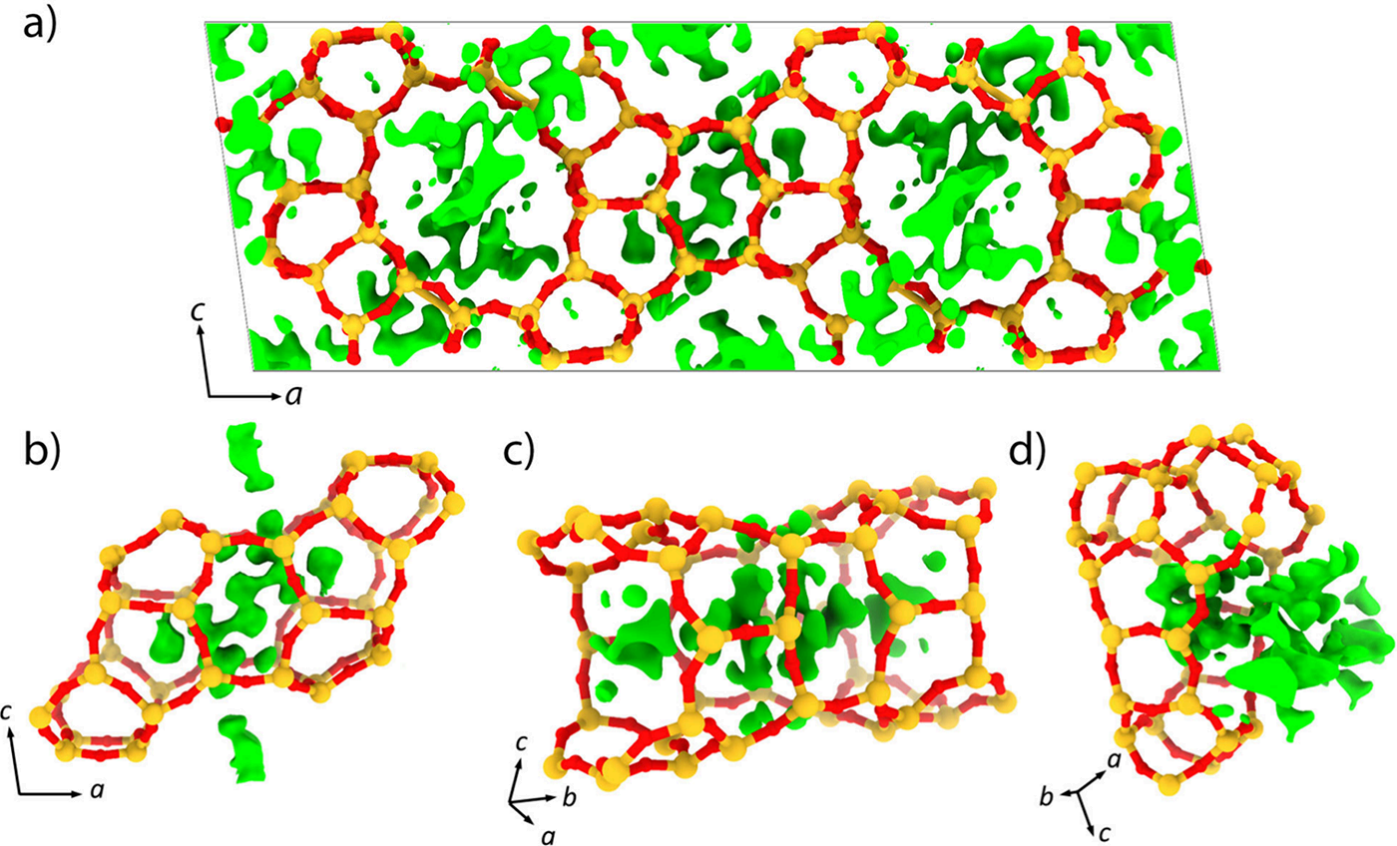
Framework of as-made EMM-59 superimposed with the difference electrostatic potential map shown at the 2σ level in green. (a) Distribution of the residual potential peaks in the unit cell indicating the locations of the OSDAs. The OSDAs were found to be localized in the (b) [4^4^.5^12^.6^2^.10^4^] cavity, (c) 12A channel, and (d) [4^2^.5^6^.6^5^.10.12] cavity.

Positive electrostatic potential peaks are observed in the difference map within the pore system (Figure 4), which correspond to the OSDAs. However, the peaks are too diffuse to be assigned to individual atoms of the OSDAs. This can be attributed to the highly flexible nature of the OSDAs. Nevertheless, it was possible to identify three symmetry-independent locations of the OSDAs from three sets of positive peaks in the difference map. One is found in the [4^4^.5^12^.6^2^.10^4^] cavity at the center of the unit cell extending into the 12A channels (Figure 4a). This cavity acts as a junction connecting the 12A and 12B channels via the four 10-ring windows (Figure 4b). The second set extends along the 12A channel (Figure 4c). The last is in the [4^2^.5^6^.6^5^10.12] cavity and extends into the 12B channel through the 12-ring window (Figure 4d). There are two [4^4^.5^12^.6^2^.10^4^] cavities, four [4^2^.5^6^.6^5^.10.12] cavities, and two 12A channels in each unit cell, which corresponds to eight OSDAs per unit cell, consistent with the number identified from the TG-DTA data (7.7 OSDA, see Figure S6).

### Structure Determination and Refinement of Calcined EMM-59

**Figure 5:**
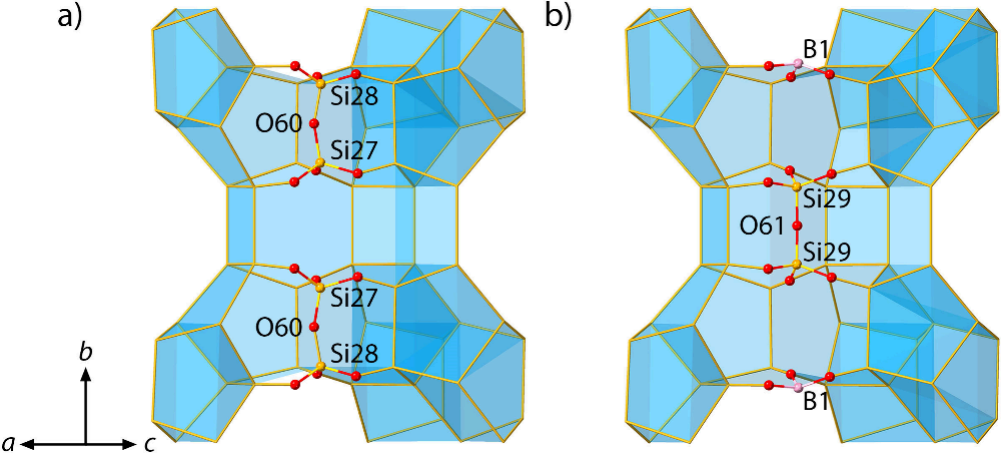
Refinement against 3D ED data of calcined EMM-59 shows the presence of two configurations (a) with full tetrahedral coordination and (b) combined trigonal and tetrahedral coordination where the boron atoms have adopted a trigonal environment.

3D ED data of the calcined sample were collected at room temperature using a cumulative electron fluence of 6 e^–^/Å^2^. The 3D ED data could be indexed using a monoclinic unit cell with *a* = 41.66(12) Å, *b* = 19.45(7) Å, *c* = 15.19(6) Å, and β = 98.48(43)°. The initial structure solution resulted in a fully tetrahedral framework structure with the same topology as that of as-made EMM-59. However, positive peaks were observed in the difference Fourier map in the vicinity of the T27–O60–T28 pairs (Figure S7). One residual peak was found near the T28 site, which was refined to a boron atom (B1 site) with 38.6% occupancy in trigonal BO_3_ coordination (Figure 5a). The absence of the residual peak in the as-made form suggests that trigonal BO_3_ originates from the conversion of tetrahedral BO_4_ at the T28 site upon calcination. Since the T28 site in the calcined form refined exclusively to silicon with 62.4% occupancy, all tetrahedral boron atoms at the T28 site in the as-made form transformed to trigonal BO_3_ after calcination to remove the OSDAs. Moreover, three peaks were found between the T27 sites forming a T–O–T bridge (Figure S7, Figure 5b). Refinements led to occupancies of 0.62 (Si27, Si28, and O60) and 0.38 (B1, Si29, and O61), indicating the presence of two complementary parts. Without boron, the two Si27–O60–Si28 pairs are present, and with boron, the B1 and Si29–O61–Si29 bridges are present. The model describes the case in which both pairs contain boron. When only one pair contains boron, it is most likely that one of the Si29 sites would turn into a Q3 silicon species connected to a hydroxyl group at the O61 site. The refinement converged, and the R_1_ value improved from 15.95% to 15.51% after modeling the trigonal site and the T–O–T bridge (see details in Tables S4–6). Solid-state ^11^B and ^29^Si NMR (Figures S8 and S9) show that the as-made EMM-59 is fully tetrahedral, whereas in the calcined form a significant portion of boron atoms adopt trigonal coordination, consistent with the results from the 3D ED refinement.

Refinements against the data from the calcined material confirm the incorporation of boron into four additional T sites: T15, T21, T25, and T26. However, the occupancies of boron at these sites show a large variation between data sets, as detailed in Table S7, preventing accurate quantification of their contents. All five T-sites that contain boron belong to 4-rings, an observation consistent with earlier studies of boron location using NMR and X-ray diffraction techniques (Tables S8–16).^10,11,14,17,32^ T28, which has the highest boron content, is shared by three 4-rings and exhibits a tetrahedral geometry with average T–O distances of 1.48 Å, which is consistent with the typical B–O distances of 1.47 Å.^33^ Based on the refinement against as-made 3D ED data, T28 is observed to be surrounded by one diquaternary OSDA. Notice that T21, T26, and T28, all containing B, belong to the same 4-ring. No boron is, however, found at T27 of the same 4-ring despite the similar framework environment as T28. This can be attributed to the lower accessibility of the charge-balancing ammonium headgroup. These observations demonstrate the preferred location of boron atoms in a 4-ring, where the OSDA provides further stability of the negatively charged BO_4_^–^ tetrahedra. When the site becomes trigonal upon calcination, three 4-rings become dismantled, likely relieving strain present in the framework. The synergistic effect of boron in creating otherwise geometrically strained locations and in providing charge balance for the ammonium head groups explains the combined structure-directing ability of the OSDA and boron in the formation of EMM-59.

### STEM Imaging

**Figure 6:**
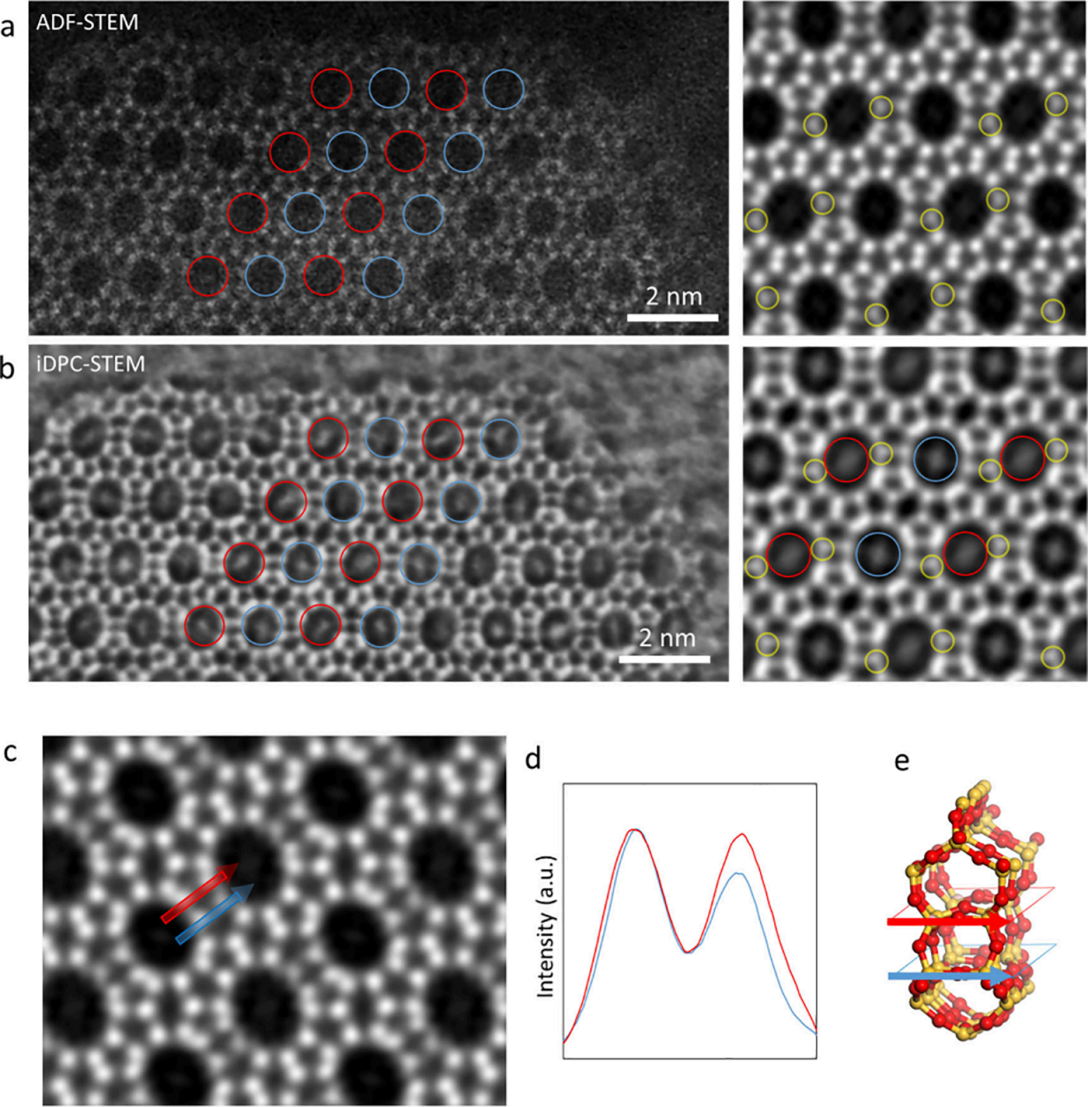
(a) ADF-STEM and (b) iDPC-STEM images of as-made EMM-59 acquired along the [010] direction. The ADF-STEM image reveals the two types of 12-ring channels running along the *b*-axis of 12A (blue) and 12B (red). T-sites rich in boron are shown with lower contrast (marked by yellow circles). In the iDPC-STEM images, the OSDA molecules are visible inside the 12-ring channels. The orientations of the OSDA molecule are different in the 12A and 12B channels. (c) Lattice averaged projected potential map based on an ADF-STEM image acquired along the [010] direction of as-made EMM-59. (d) Line profile along two pairs of T atom columns with the same number of T and O atoms reveal the preferred location of boron in one of the columns. (e) Illustration of the location of the line profile in the structure.

To study the local structure of EMM-59, STEM images were acquired along the direction of the large-pore channels corresponding to the [010] direction. ADF and iDPC-STEM images were acquired simultaneously from the as-made material. The ADF-STEM imaging revealed the two different types of 12-ring channels running along the *b*-axis, 12A and 12B (Figure 6a). The contrast of the ADF-STEM image scales with the atomic number, which enables differentiation of boron-rich columns from columns with the same number of T and oxygen atoms (Figure 6c-e). The location of the boron-rich columns agrees with the boron-rich site (B1) located from refinement against 3D ED data and can be confirmed by simulations of STEM images (Figure S10). This illustrates the localization of potential acid sites from the ADF-STEM images.

The contrast of the iDPC-STEM image, which is more sensitive to light elements, reveals the OSDAs inside the 12-ring channels of the as-made material, unlike images from the calcined material (Figure S11). The iDPC-STEM imaging shows different configurations of the OSDAs channel-by-channel. The contrast observed within the 12A and 12B channels shows distinct configurations of the OSDAs. In the 12A channels, which lack boron-rich sites, the OSDAs are situated near the center of the channel (Figure 6b). On the other hand, in the 12B channels, the molecules often appear elongated toward the boron-rich sites. Notably, various configurations of the OSDA can be seen within the pores of the same framework environment, further showing the flexible nature of the OSDAs. The flexible nature of the aliphatic OSDAs likely allows them to adopt multiple configurations while keeping the head groups at a reasonable distance of ∼4.5 Å from the O next to B sites. The distinct contrast observed in the two types of 12-ring channels demonstrates the ability of iDPC-STEM to differentiate configurations of the OSDA molecule. This variability underscores the challenge of accurately determining the OSDA positions from the diffraction data.

## Conclusions

A novel borosilicate zeolite, EMM-59, was synthesized using a diquaternary ammonium salt as the OSDA. EMM-59 has a monoclinic structure with a 3D 12 × 10 × 10-ring channel system, which was determined using 3D ED. Electron diffraction can be used to refine the site occupancies for element pairs with substantial difference in their scattering factors such as boron and silicon. Least-squares refinement against the 3D ED data acquired from as-made and calcined EMM-59 shows that one of the tetrahedral sites partially turns into a trigonal coordination upon calcination. This reveals a highly localized boron site, which is further confirmed using ADF-STEM images.

Locating a flexible OSDA disordered in the pores of zeolite is intrinsically challenging. Scanning transmission electron microscopy imaging herein provided local insights, revealing that the OSDA molecules have different orderings in the two types of 12-ring channels. Furthermore, the STEM images reveal a significant variation in the orientation of the OSDA within pores of the same environment, further pinpointing the difficulty to locate the atomic position of the OSDA molecules by 3D ED. The combination of 3D ED and STEM analysis presented herein shows that boron is mainly observed in sites that have a strained geometry often in the junctures of several 4-rings and at the same time in the vicinity of the OSDA molecules to stabilize the BO_4_^–^ tetrahedron, which is further confirmed by the altered coordination of boron atoms upon the removal of the charge balancing OSDAs.

## Experimental Section

### Synthesis of OSDAs

The synthesis of 1,1-(2,2-(cyclopentane-1,1-diyl)bis(ethane-2,1-diyl))bis(1-ethylpyrrolidinium) and 2,2-(cyclopentane-1,1-diyl)bis(*N*,*N*-diethyl-*N*-methylethan-1-aminium) hydroxide used as OSDAs is detailed in the Supporting Information.

### Zeolite Synthesis

EMM-59 was synthesized using 2,2-(cyclopentane-1,1-diyl)bis(*N*,*N*-diethyl-*N*-methylethan-1-aminium) as the OSDA. KOH was used together with OSDA-OH from a gel with the following ratios: OH^–^/Si= 0.3, Si/B = 20, K/Si = 0.1, and H_2_O/Si = 30. 3.4 g of an 18.4 wt % solution of 2,2-(cyclopentane-1,1-diyl)bis(*N*,*N*-diethyl-*N*-methylethan-1-aminium) hydroxide was added to 2.62 g of deionized water and mixed well using a magnetic stirrer. To this mixture 3.74 g of 30 wt % silica in water (AERODISP W 7330 N), 1.7 g of 3.47 wt % boric acid and 0.6 g of 17.5 wt % KOH were added and stirred for 30 min. The mixture was placed in a 23 mL Teflon liner which was sealed within a stainless-steel digestive reactor. The reactor was placed in a convection oven with a tumbling rotisserie (30 rpm) at 150 °C. After 14 days, the product was formed. EMM-59 could also be synthesized with 1,1-(2,2-(cyclopentane-1,1-diyl)bis(ethane-2,1-diyl))bis(1-ethylpyrrolidinium) as the OSDA with gel composition OH^–^/Si = 0.30, Si/B = 20, KOH/Si = 0.10, and H_2_O/Si = 30. After 9 days of heating at 150 °C, the product was formed.

### Characterization

Liquid-state ^1^H and ^13^C NMR spectra were collected on a Bruker Spectron Spin 400. Solid-state ^11^B and ^29^Si MAS NMR spectra were collected on an 11.7 T Varian Infinity Plus 500 (IP-500) spectrometer corresponding to Larmor frequencies of 160.2 and 99.2 MHz, respectively. Elemental analyses were done by ICP at Galbraith Laboratories. Thermogravimetric analyses (TGA) were done with a Mettler Toledo TGA/DSC1 using the following program ramp in air from 30 to 800 °C at 4 °C/min. TGA results showed 17.33 wt % loss due to the organic decomposition. SEM images were collected on a JEOL JSM-7000F field emission scanning electron microscope. The powder XRD data were collected on a Bruker Endeavor Gadds system. Data were collected with CuKα radiation from 4 to 50° 2theta using a step size of 0.02° 2theta and a count time of 0.15 s per step.

### Calcination of As-Made Sample

The as-made sample was purged with N_2_ for 2 h at room temperature before ramping to 400 °C at 4 °C/min. The final temperature was maintained for 15 min before switching N_2_ to air. The temperature was ramped to 540 °C at 4 °C/min and maintained at the temperature for 3 h.

### Three-Dimensional Electron Diffraction (3D ED) Data Collection

The samples were crushed, dispersed in absolute ethanol, treated by sonication for 10 min, and then transferred onto Quantifoil grids (R2/2 + 2 nm carbon Cu 300 mesh grid, Quantifoil Micro Tools). Grids with as-made EMM-59 were plunged into liquid ethane and subsequently cooled in liquid nitrogen. The grids were transferred via an autoloader to Krios G3i TEM with an XFEG electron source (Thermo Fisher Scientific) operated at 300 kV. A parallel beam was created with a combination of C2 and C3 lenses using a 20 μm C2 aperture operating in microprobe mode. The microbeam radius was adjusted to produce a flux of ∼0.0025 e^–^/Å^2^·s. The 3D ED data were obtained using the continuous rotation electron diffraction (cRED) technique. Each data set was collected with an exposure time of 0.5s for each diffraction pattern with a Ceta-D detector (Thermo Fisher Scientific) while constantly rotating at 0.3°/s for a total of 60° using EPU-D (Thermo Fisher Scientific), corresponding to a total fluence of 0.5 e^–^/Å^2^ per data set. 3D ED data for calcined EMM-59 were collected on JEOL JEM-2100 operated at 200 kV by continuously tilting the crystal at 0.23°/s. The flux used was calibrated to be ∼0.0111 e^–^/Å^2^·s. The cumulative fluence for rotation data sets was up to 6.0 e^–^/Å^2^ depending on the stage tilt. The electron diffraction patterns were collected on a high-speed hybrid Timepix Quad camera (Amsterdam Scientific Instruments) controlled through Instamatic software.^34^

### Data Processing and Structural Analysis

The 3D ED data were processed through XDS and merged with XSCALE.^35^ All framework atoms were found *ab initio* using SHELXT.^36,37^ Refinement against 3D ED data was performed using SHELXL^38^ through Olex2^39^ with atomic scattering factors for electrons.^40^ Bond geometries were examined using zeoTsites^41^ and ToposPro.^42^

### Scanning Transmission Electron Microscopy

The EMM-59 samples were embedded into an epoxy resin and sectioned by ultramicrotomy into sections of ∼50 nm thickness. The sectioned samples were dried in a vacuum at 180 °C for 3 h prior to data collection to remove adsorbed water molecules in the pores and increase the zeolite stability. The samples were imaged at 300 kV using a double aberration-corrected Themis Z TEM (Thermo Fisher Scientific). The images were acquired using a beam current of 9 pA, a convergence angle of 16 mrad, a dwell time of 5 μs, and a pixel size of 37 pm. Integrated differential phase contrast (iDPC) images were obtained by using a segmented annular dark-field detector covering a collection angle of 6–24 mrad. The ADF-STEM images were obtained with a collection angle of 25–153 mrad. A high-pass filter was applied to the iDPC images to reduce the low-frequency contrast. ADF-STEM simulations were performed using Dr. Probe with a convergence angle of 16 mrad and collection angles of 25–100 mrad.^43^

## Supporting Information Available

Experimental section, synthesis details, characterization details and crystallographic information (PDF)
