# Supplementary material for: Localized Boron Sites in Large Pore Borosilicate Zeolite EMM-59 Determined by Electron Crystallography
Source: J Am Chem Soc. 2024 Dec 9;146(50):34916–23. doi: 10.1021/jacs.4c14478 (PMC11664499; doi:10.1021/jacs.4c14478)
Supplement: Supplementary file 1 — ja4c14478_si_001.pdf [file ja4c14478_si_001.pdf]

Localized Boron Sites in Large Pore Borosilicate Zeolite EMM-59 Determined by Electron Crystallography

Jung Cho[1], Elina Kapaca[1], Bin Wang[1], Ross Mabon[2], Hilda Vroman[2], Xiaodong Zou[1], Allen W. Burton[2,*], and Tom Willhammar[1,*]

[1]Department of Materials and Environmental Chemistry, Stockholm University, SE-106 91 Stockholm, Sweden

[2]Corporate Strategic Research, ExxonMobil Research & Engineering Co. Inc, 1545 Route 22 East, Annandale, New Jersey 08801, USA

*Corresponding authors: allen.w.burton@exxonmobil.com, tom.willhammar@mmk.su.se

Content

Synthesis of organic structure directing agents (OSDAs)	3
Figure S1. Electron scattering factors of common T atoms, B, Si, Al, and Ge.	6
Figure S2. PXRD pattern of the calcined EMM-59	7
Figure S3. Scanning electron microscope (SEM) image of as-made EMM-59	8
Figure S4. Column based building scheme of ZSM-5 and ZSM-11.	9
Figure S5. Different combinations of 2x2 matrices of the building scheme of EMM-59	10
Figure S6. TGA from as-made EMM-59	11
Figure S7. Difference electrostatic potential map of calcined EMM-59	12
Figure S8. 29Si Solid-state NMR from (green) as-made and (red) calcined EMM-59.	13
Figure S9. 11B solid-state NMR	14
Figure S10 Simulated ADF-STEM images	15
Figure S9. (a) ADF-STEM and (b) iDPC-STEM images of calcined EMM-59.	16
Table S1. Unit cell parameters of as-made EMM-59 crystal datasets reported by XDS.	17
Table S2. Data merging statistics per resolution bin for as-made EMM-59 reported by XSCALE.	18
Table S3. Experimental and refinement details for the cRED data of as-made EMM-59.	19
Table S4. Unit cell parameters of calcined EMM-59 crystal datasets reported by XDS.	20
Table S5. Data merging statistics per resolution bin for calcined EMM-59 reported by XSCALE.	21
Table S6. Experimental and refinement details for the cRED data of calcined EMM-59.	22
Table S7. Point symbol, boron occupancy and average ∠TOT of calcined EMM-59.	23
Table S8. Point symbol, boron occupancy and average ∠TOT of as-made SSZ-53 (SFH).3	24
Table S9. Point symbol, boron occupancy and average ∠TOT of as-made SSZ-55 (ATS).3	25

Table S10. Point symbol, boron occupancy and average ∠TOT of as-made SSZ-56 (SFS).3 26

Table S11. Point symbol, boron occupancy and average ∠TOT of as-made SSZ-58 (SFG).3 27

Table S12. Point symbol, boron occupancy and average ∠TOT of as-made SSZ-59 (SFN).3 28

Table S13. Point symbol, boron occupancy and average ∠TOT of as-made SSZ-82 (SEW).4 29

Table S14. Point symbol, boron occupancy and average ∠TOT of as-made SSZ-87 (IFW).5 30

Table S15. Point symbol, boron occupancy and average ∠TOT of calcined EMM-26 (EWS).6 31

Table S16. ∠B-O-Si and B-O in borosilicates 32

Synthesis of organic structure directing agents (OSDAs)

Several OSDA molecules can be used to prepare EMM-59. The procedure for preparing 1,1-(2,2-(cyclopentane-1,1-diyl)bis(ethane-2,1-diyl))bis(1-ethylpyrrolidinium) and 2,2-(cyclopentane-1,1-diyl)bis(N,N-diethyl-N-methylethan-1-aminium) are as follows:

Spirocyclopentyl Derivative:
2,2'-(cyclopentane-1,1-diyl)bis(1-(pyrrolidin-1-yl)ethan-1-one):

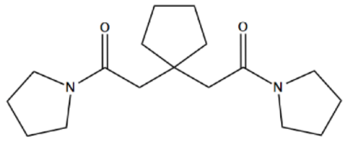

2,2'-(cyclopentane-1,1-diyl)bis(1-(pyrrolidin-1-yl)ethan-1-one)

To a flame dried 500 mL round bottom flask with stir bar was added 100 mL of anhydrous tetrahydrofuran. 3,3-tetramethyleneglutaric anhydride (10.0 g, 59.5 mmol) was added to the flask and the flask was purged with nitrogen. Pyrrolidine (12.69 g, 178.4 mmol, 3 eq) was added via syringe and the reaction mixture was refluxed at 95 °C for 1 hour. The heat was removed, and the reaction cooled to room temperature. Pyridine (15.53 g, 196.4 mmol, 3.3 eq) was added via syringe and the reaction mixture was cooled to 0 °C. Propylphosphonic anhydride solution (50% by wt in EtOAc, 75.73 g, 119.0 mmol, 2 eq) was slowly poured in from a beaker (anhydride solution is very viscous) and reaction stirred under nitrogen in the ice bath overnight (ice allowed to melt). 200 mL of 10% aqueous HCl was added to the reaction mixture. This solution was stirred vigorously for 24 hours. The reaction mixture was transferred to a 500 mL separatory funnel and extracted 3 times with 75 mL of methylene chloride. Organics were dried with sodium sulfate, gravity filtered into a pre-weighed round bottom, and solvent was removed under vacuum to yield an oil (16.87 g, 57.7 mmol, 97% yield). The product was pure enough for the next step without further purification. 1H NMR (400MHz, CDCl3) δ ppm: 3.30 (m, 8H), 2.50 (s, 4H), 1.78 (m, 4H), 1.68 (m, 4H), 1.60 (m, 4H), 1.47 (m, 4H).

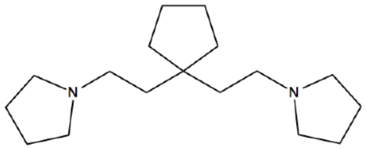

1,1'-(cyclopentane-1,1-diylbis(ethane-2,1-diyl))dipyrrolidine

1,1'-(cyclopentane-1,1-diylbis(ethane-2,1-diyl))dipyrrolidine:

To a flame dried 1000 mL 3 necked round bottom flask with stir bar was added 400 mL of anhydrous tetrahydrofuran. Slowly, lithium aluminum hydride powder (5.00 g, 131.8 mmol, 2.5 eq) was added to the stirring solution. Separately, the diamide (15.40 g, 52.70 mmol) was dissolved in 100 mL of anhydrous tetrahydrofuran and transferred to a flame dried liquid addition funnel. The diamide solution was slowly dripped into the lithium aluminum hydride suspension. Once the diamide solution was completely added, the rubber stoppers were replaced with glass stoppers and the reaction mixture refluxed under nitrogen for 2 hours before stirring at room temperature overnight. The reaction vessel was uncapped, and 5.00 mL of deionized water was added to a liquid addition funnel and added dropwise to the stirring reaction mixture. Following this, 5.00 mL of 10% aqueous NaOH was added to the liquid addition funnel and added dropwise to the stirring mixture. Next, 15.00 mL of deionized water was dripped into the stirring mixture and the mixture was stirred at room temperature for 1 hour. The reaction mixture was filtered through a celite pad and the solid aluminum hydroxide salts were washed 3 times with 50 mL of diethyl ether. The filtrate was dried with sodium sulfate, gravity filtered into a pre-weighed round bottom flask, and solvent was removed under vacuum to yield a colorless oil (12.54 g, 47.43 mmol, 90% yield). The product was pure enough for the next step without further purification. 1H NMR (400MHz, CDCl3) δ ppm: 2.48 (m, 8H), 2.43 (m, 4H), 1.77 (m, 8H), 1.58 (m, 4H), 1.50 (m, 4H), 1.40 (m, 4H).

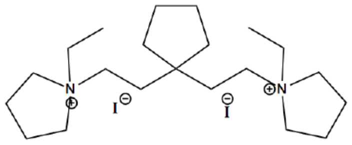

1,1'-(cyclopentane-1,1-diylbis(ethane-2,1-diyl))bis(1-ethylpyrrolidin-1-ium) iodide

1,1'-(cyclopentane-1,1-diylbis(ethane-2,1-diyl))bis(1-ethylpyrrolidin-1-ium) iodide:

The diamine from the previous procedure (12.54 g, 47.43 mmol) was dissolved in 100 mL of acetone in a 250 mL round bottom flask with stir bar. Iodoethane (36.99 g, 237.15 mmol, 5 eq) was added via syringe and the reaction stirred at room temperature while loosely capped for 72 hours. The very cloudy reaction mixture was filtered through a medium frit funnel and the resulting white solid was washed 3 times with 20 mL of acetone and dried to afford pure product (21.31 g, 37.0 mmol, 78% yield). 1H NMR (400MHz, D2O) δ ppm: 3.25-3.40 (m, 8H), 3.05-3.20 (m, 8H), 2.00 (m, 8H), 1.60 (m, 4H), 1.48 (m, 4H), 1.35 (m, 4H), 0.80 (t, 6H).

1,1'-(cyclopentane-1,1-diylbis(ethane-2,1-diyl))bis(1-ethylpyrrolidin-1-ium) iodide was

converted to its hydroxide form by dissolving it in water and adding it to 700 mL of Dowex LC NG hydroxide exchange resin. After contacting the exchange resin overnight, the resin was removed by filtration and washed with deionized water. The aqueous fractions were then combined and concentrated under reduced pressure at about 60 °C. The hydroxide concentration of this aqueous solution was 16.6 wt% as determined by titration with a standard solution of 0.1 N HCl.

A similar procedure was used to prepare 2,2-(cyclopentane-1,1-diyl)bis(N,N-diethyl-N-methylethan-1-aminium) except 1) diethylamine was used instead of pyrrolidine in the first step and 2) iodomethane was used as an alkylating agent instead of iodoethane in the final step to prepare the quaternary ammonium molecule.

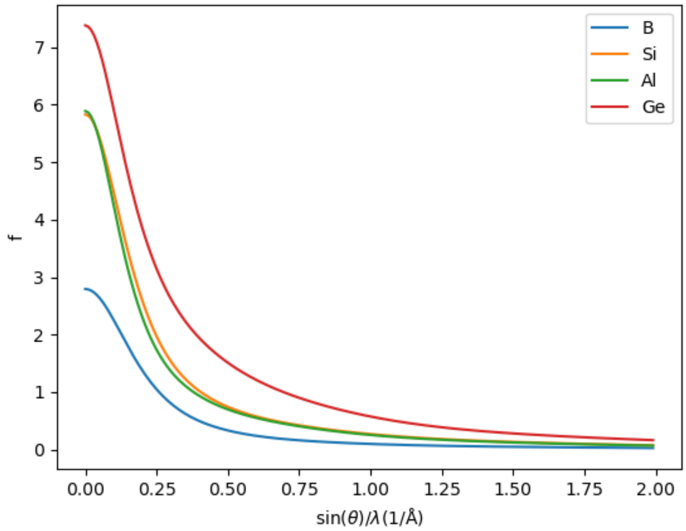

Figure S1. Electron scattering factors of common T atoms, B, Si, Al, and Ge.

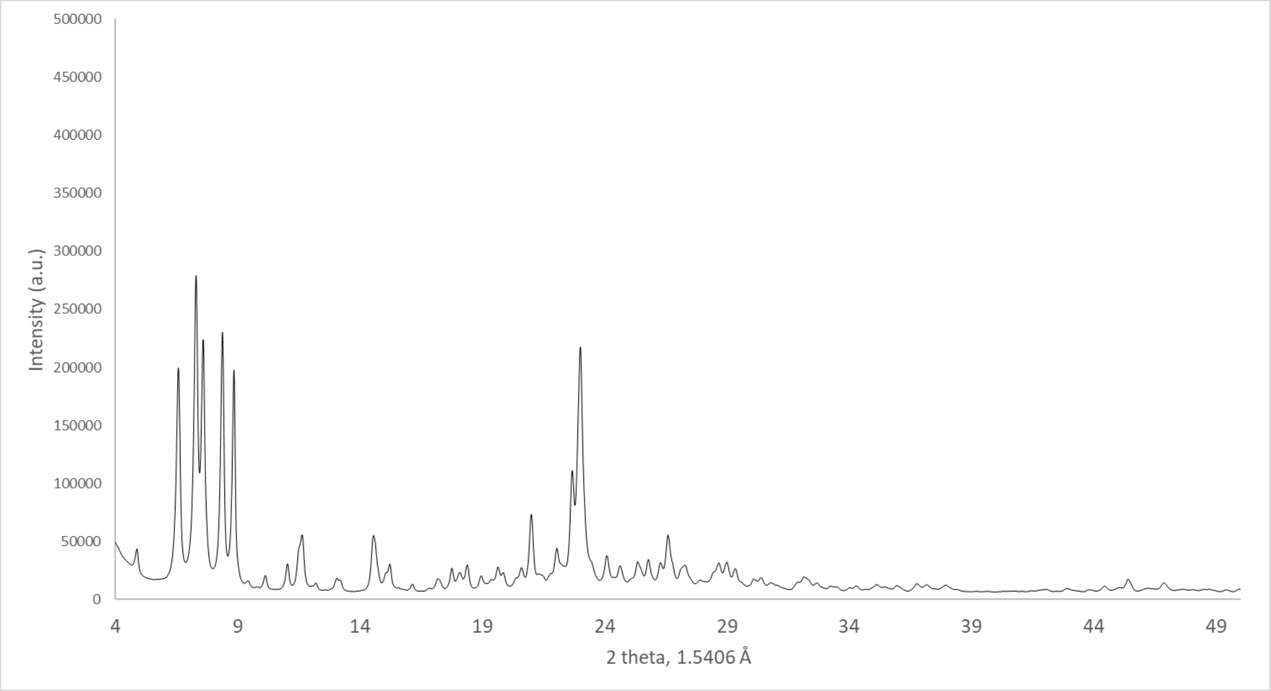

Figure S2. Powder X-ray diffraction pattern of calcined EMM-59.

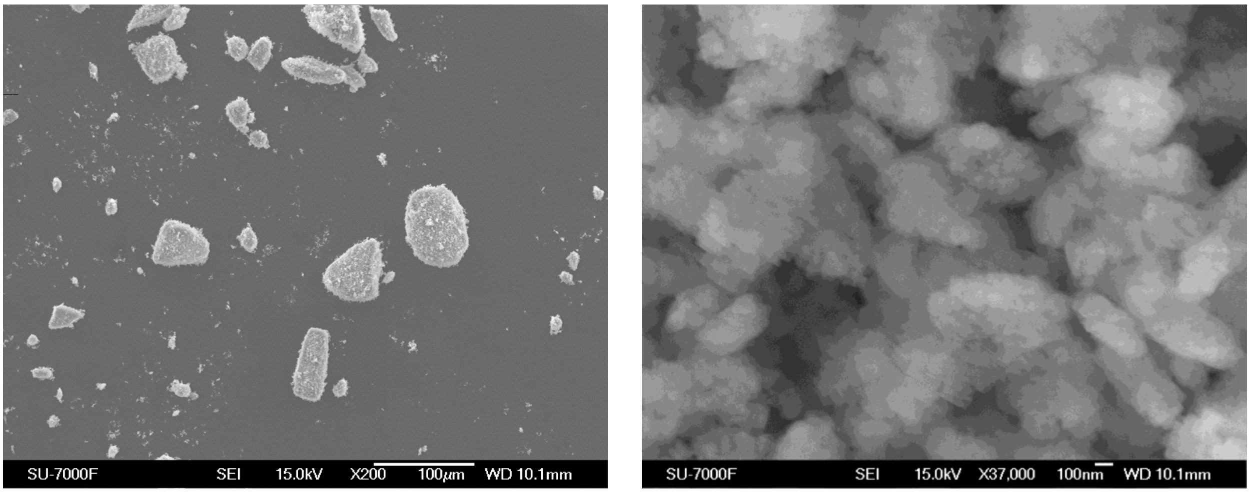

Figure S3. Scanning electron microscope (SEM) image of as-made EMM-59

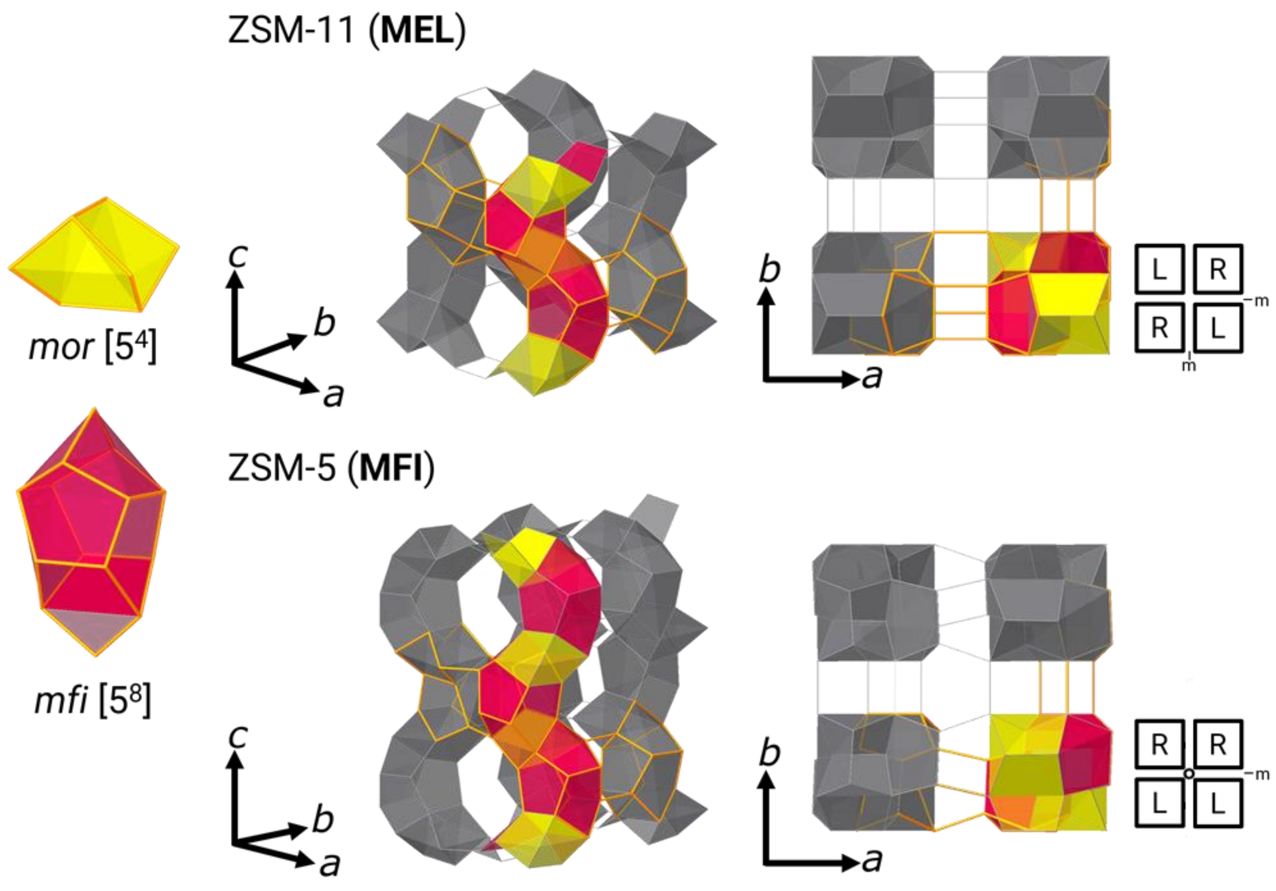

Figure S4. Column-based building scheme of ZSM-5 and ZSM-11. *mor* and *mfi* cages are colored in yellow and red, respectively. Butterfly units located along *b* and *a*-axes are traced with yellow bonds. Each enantiomorphic column can be extracted by slicing through the 6-rings of the butterfly units.

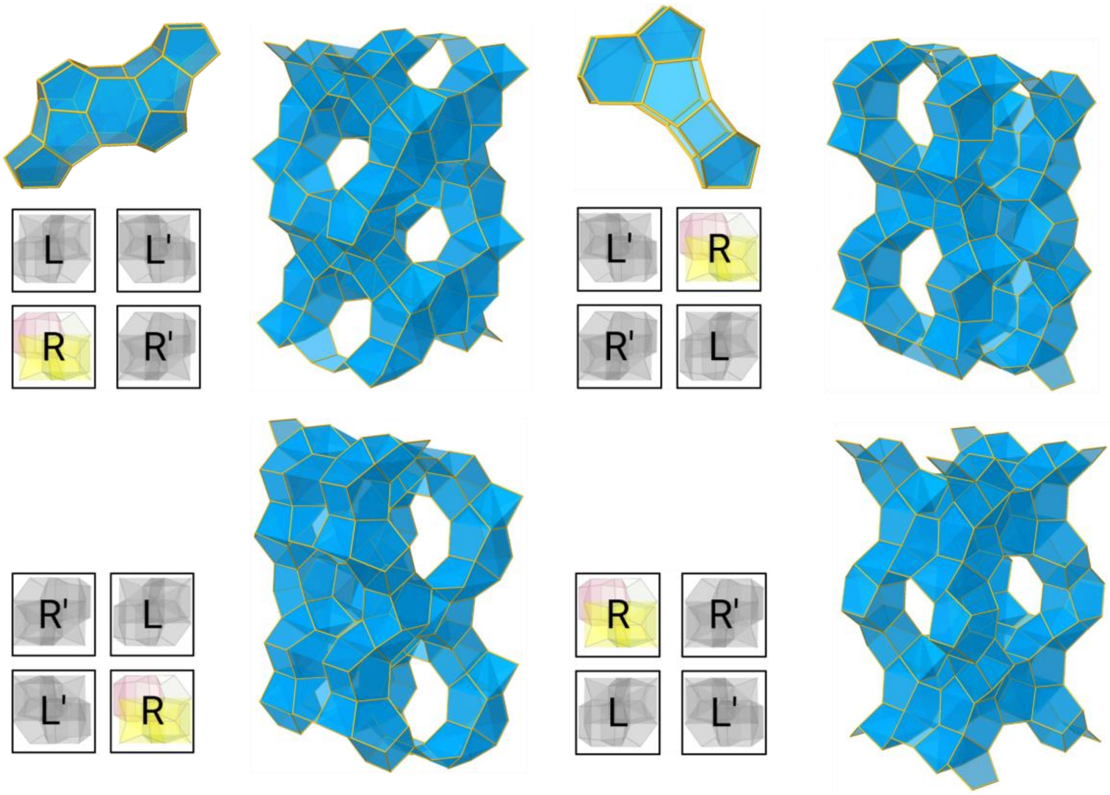

Figure S5. Different combinations of 2x2 matrices of the building scheme of EMM-59 yields different channel intersecting cavities.

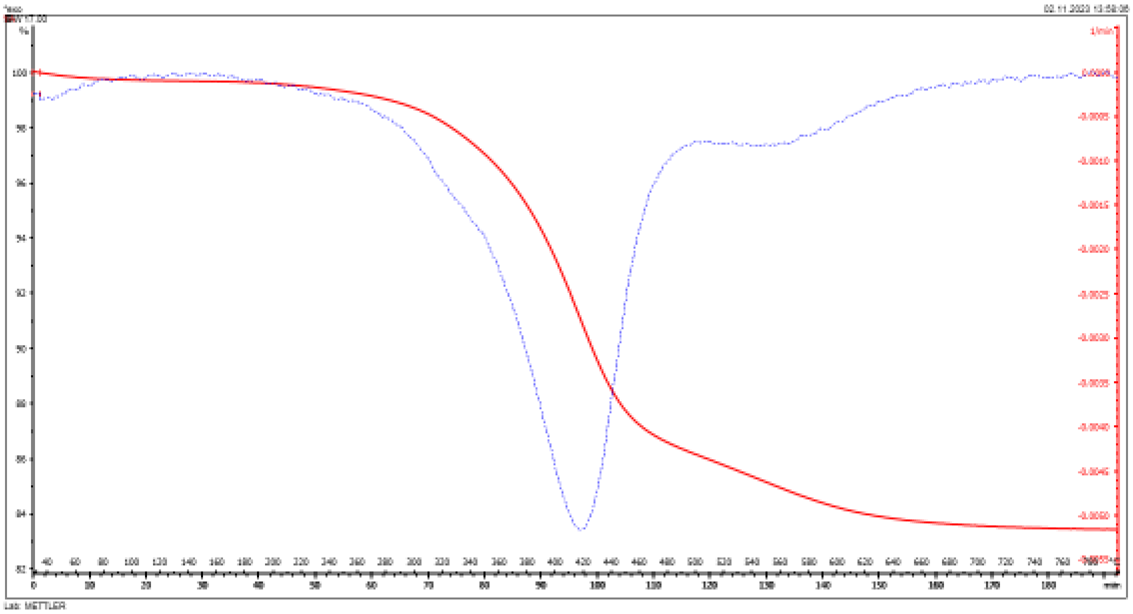

Figure S6. TGA obtained from as-made EMM-59.

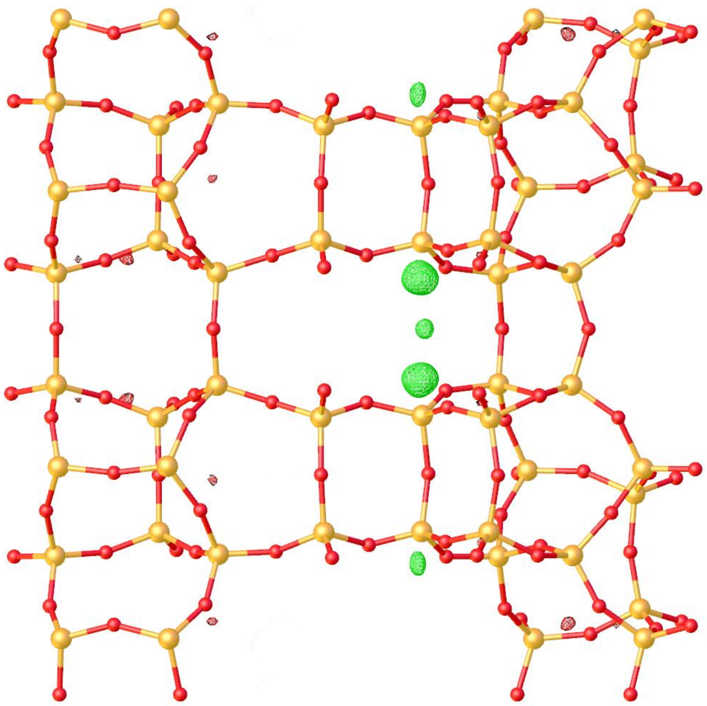

Figure S7. Difference electrostatic potential map of calcined EMM-59 shows the trigonal B sites (green bottom and top) as well as the new T-O-T bridge (green middle).

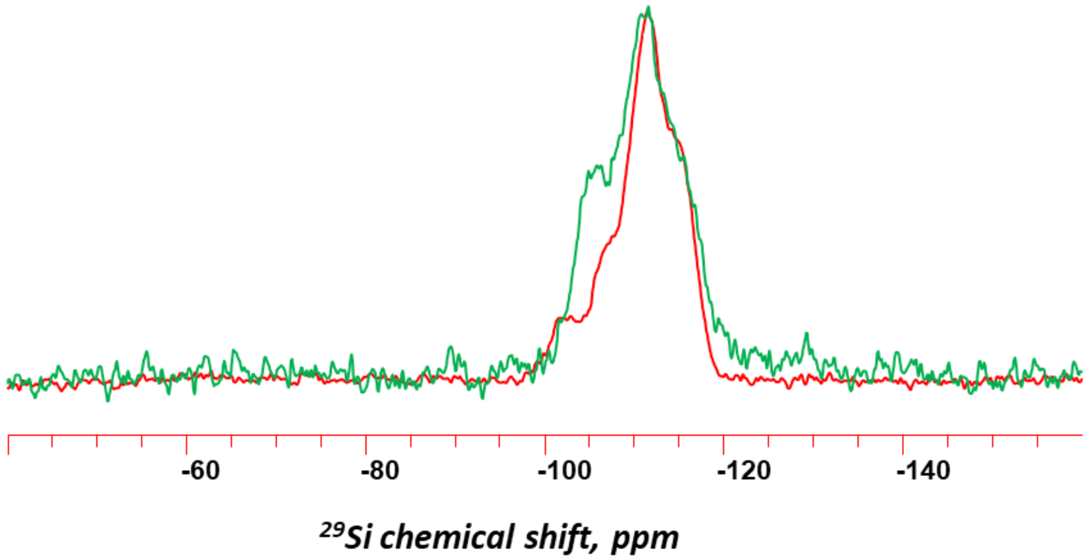

Figure S8 ^{29}Si Solid-state NMR from (green) as-made and (red) calcined EMM-59.

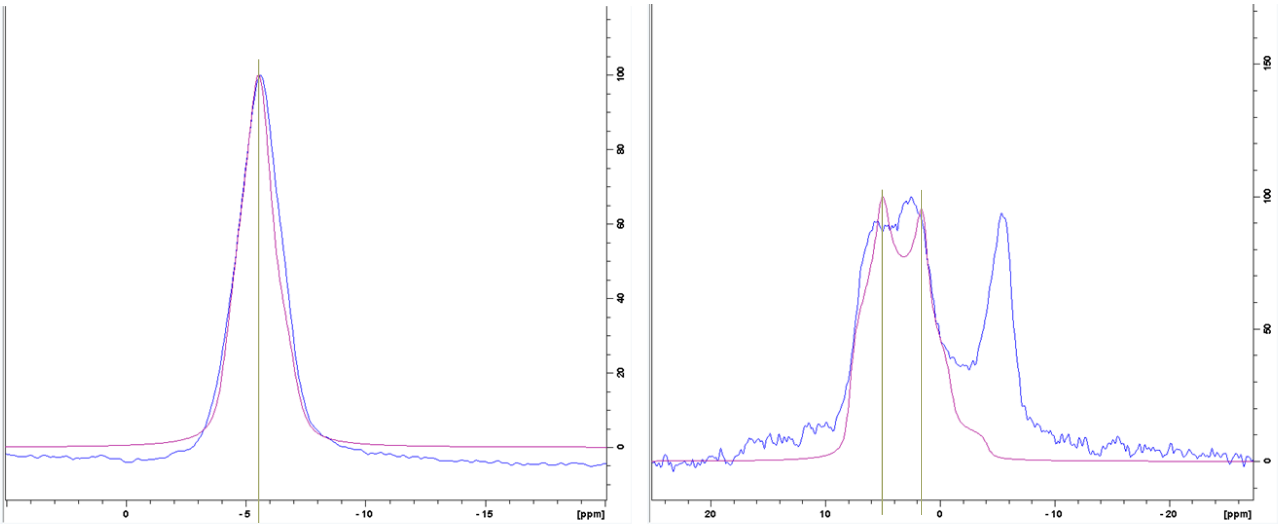

Figure S9. ^{11}B solid-state NMR from (a) as-made and (b) calcined EMM-59. The peaks at -5.5 ppm corresponds to tetrahedral B and the peaks at ~5 ppm to trigonal B.

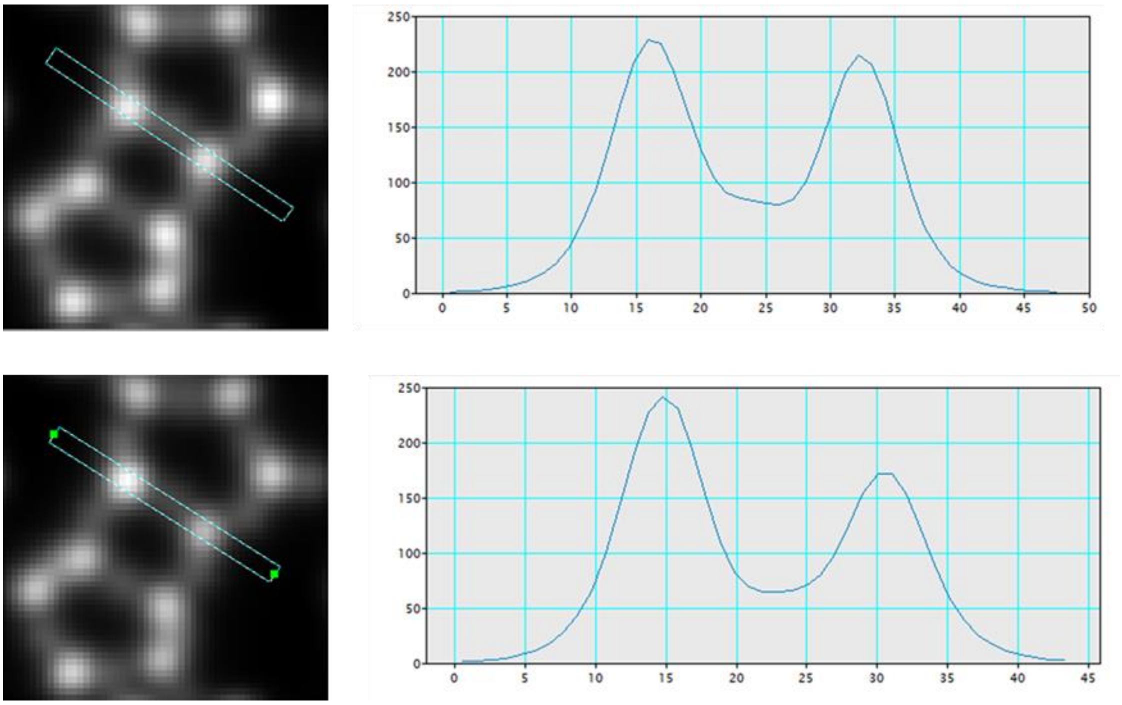

Figure S10 Simulated ADF-STEM images based on a fully siliceous structure of as-made EMM-59 (top) and a structure with 38% B and 62% Si for the T28-site (bottom). The line profile across the two columns shows the expected difference in contrast provided by the incorporation of boron.

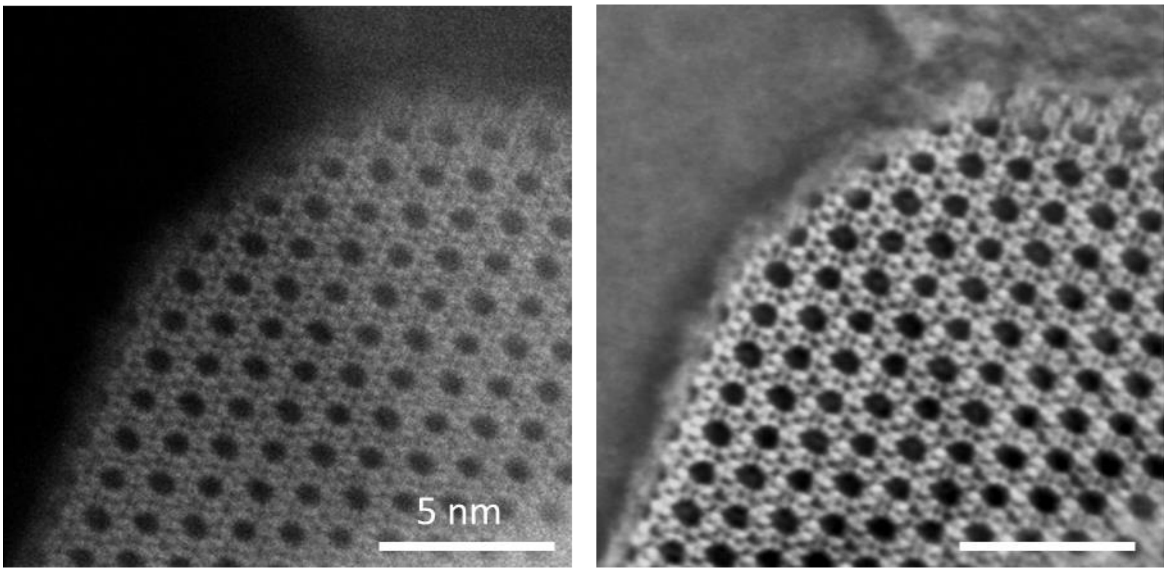

Figure S11. (a) ADF-STEM and (b) iDPC-STEM images of calcined EMM-59.

Table S1. Unit cell parameters of as-made EMM-59 crystal datasets reported by XDS. Data were integrated using the Laue group 2/*m*. Average unit cell parameters were determined to be a = 42.44(18), b = 19.90(7), c = 15.40(9) Å, β = 97.98(45)°.

**Dataset**	***a* (Å)**	***b* (Å)**	***c* (Å)**	**β (°)**
1	42.50	19.87	15.34	98.0
2	42.35	19.78	15.38	97.7
3	42.12	19.99	15.25	97.2
4	42.41	19.95	15.45	98.0
5	42.62	19.86	15.41	98.5
6	42.64	19.92	15.54	98.5

Table S2. Data merging statistics per resolution bin for as-made EMM-59 reported by XSCALE.

Resolution Limit	Observed Reflections	Unique Reflections	Completeness (%)	R_{merge} (%)	R_{meas} (%)[1]	I/σ(I)	$CC_{1/2}$ (%)[2]
3.89	1773	134	89.9	15.3	15.8	12.97	99.7*
2.75	3318	245	95.3	14.8	15.4	10.58	99.0*
2.25	4289	294	91.6	24.2	25.1	8.44	99.6*
1.95	5308	372	94.4	29.7	30.7	5.91	99.5*
1.74	5689	399	94.3	43.9	45.4	4.94	98.0*
1.59	6718	462	95.1	56.3	58.1	4.43	96.2*
1.47	6985	481	95.2	48.6	50.1	4.48	99.2*
1.38	7638	529	95.1	70.4	72.7	3.44	98.2*
1.30	8467	569	95.6	116.9	120.6	2.67	91.9*
1.23	8731	589	96.6	73.0	75.3	3.34	97.8*
1.17	9363	644	96.1	111.8	115.3	2.54	94.2*
1.12	9484	643	95.1	197.9	204.0	1.54	81.9*
Total	77763	5361	93.9	32.4	34.2	5.0	99.3*

Table S3. Experimental and refinement details for the cRED data of as-made EMM-59.

Crystal Data	
Formula	[SiO_2]
Formula Weight (g/mol)	60.08
Crystal System	Monoclinic
Space group	*C*2/*m*
a, *b*, *c* (Å)	42.44(18), 19.90(7), 15.40(9)
β (°)	97.98(45)
Volume, $Å^3$	12880(5)
Data Collection	
Detector	Ceta-D
Temperature (K)	77
Radiation (Å)	Electron, 0.01968
d_{min}, d_{max} (Å)	1.15, 18.13
Dataset (*h*, *k*, *l*)	-36→36, -17→17, -13→13
Tot., Uniq. Data, R_{int}	63317, 4405, 0.3693
Completeness, %	92.8
<*I*/σ(*I*)>	5.0
R_{meas}	0.342
$CC_{1/2}$	0.993
Tilt step per frame (°)	0.30
Exposure time per frame (s)	0.50
Refinement	
$N_{parameters}$, $N_{restraints}$	345, 0
R1 [2I/σ(I)], $N_{reflections}$	0.2032, 2162
R1 [all data], $N_{reflections}$	0.2942, 4405
GooF	1.485

Table S4. Unit cell parameters of calcined EMM-59 crystal datasets reported by XDS. Data were integrated with the Laue group 2/*m*. Average unit cell parameters were determined to be a = 41.66(12), b = 19.45(7), c = 15.19(6) Å, β = 98.48(43)°.

**Dataset**	***a* (Å)**	***b* (Å)**	***c* (Å)**	**β (°)**
1	41.80	19.60	15.21	98.4
2	41.53	19.39	15.17	98.3
3	41.67	19.36	15.24	99.1
4	41.87	19.38	15.06	98.6
5	41.62	19.49	15.25	98.4
6	41.66	19.49	15.22	99.0
7	41.60	19.42	15.20	98.4
8	41.51	19.44	15.20	97.6

Table S5. Data merging statistics per resolution bin for calcined EMM-59 reported by XSCALE.

Resolution Limit	Observed Reflections	Unique Reflections	Completeness (%)	R_{merge} (%)	R_{meas} (%)[1]	I/σ(I)	$CC_{1/2}$ (%)[2]
4.03	1425	112	99.1	17.6	18.5	13.13	99.2*
2.85	2980	200	99.5	16.3	17.0	13.47	99.5*
2.32	3870	249	100.4	17.4	18.2	12.63	98.9*
2.01	4761	292	100.0	23.5	24.4	11.01	98.6*
1.80	5537	334	99.4	17.9	18.7	10.70	98.9*
1.64	5951	349	100.3	20.6	21.3	10.27	99.6*
1.52	6782	399	99.8	21.6	22.4	10.26	99.6*
1.42	7611	440	99.8	21.6	22.4	10.10	98.9*
1.34	7756	442	100.0	20.2	20.9	9.29	99.8*
1.27	8150	458	99.8	24.8	25.7	8.71	99.1*
1.21	9050	508	100.2	26.3	27.2	8.50	99.5*
1.16	9053	507	100.2	21.3	22.0	8.90	99.0*
1.12	9970	553	99.8	24.6	25.4	8.06	99.4*
1.08	10112	561	100.2	29.5	30.4	7.00	99.2*
1.04	10653	577	99.3	43.5	44.8	5.61	98.4*
1.01	10877	592	100.9	51.6	53.2	4.44	98.6*
0.98	11721	634	100.0	59.1	60.8	4.25	96.7*
0.95	11808	632	99.5	63.5	65.4	4.05	96.7*
0.92	12070	646	100.0	59.1	60.9	3.99	98.1*
Total	150137	8485	99.2	21.1	22.4	7.34	99.4*

Table S6. Experimental and refinement details for the cRED data of calcined EMM-59.

Crystal Data	
Formula	$[Si_{0.977}B_{0.023}O_{1.993}]$
Formula Weight (g/mol)	59.56
Crystal System	Monoclinic
Space group	*C*2/*m*
a, *b*, *c* (Å)	41.66(12), 19.45(7), 15.19(6)
β (°)	98.48(43)
Volume, $Å^3$	12174(5)
**Data Collection**	
Detector	ASI TimePix
Temperature (K)	293
Radiation (Å)	Electron, 0.02508
d_{min}, d_{max} (Å)	0.92, 18.13
Dataset (*h*, *k*, *l*)	-46→46, -21→21, -16→16
Tot., Uniq. Data, R_{int}	158638, 9130, 0.2088
Completeness, %	99.20
$<I/\sigma(I)>$	7.34
R_{meas}	0.224
$CC_{1/2}$	0.994
Tilt step per frame (°)	0.23
Exposure time per frame (s)	0.50
**Refinement**	
$N_{parameters}$, $N_{restraints}$	785, 0
R1 [2I/σ(I)], $N_{reflections}$	0.1398, 7367
R1 [all data], $N_{reflections}$	0.1550, 9130
GooF	1.215

Table S7. Boron occupancies at the T15, T21, T25, T26, and T28 sites for different merged datasets.

Dataset combination	1&3	2&4	3&5	5&6	6&7	2&5	All
Completeness (%)	97.6	95.0	97.9	96.8	98.1	98.2	99.4
B occ. at T15 (%)	17	7	10	5	2	4	4
B occ. at T21(%)	4	4	11	18	11	20	7
B occ. at T25 (%)	5	2	9	12	13	13	9
B occ. at T26 (%)	4	3	9	15	4	18	5
B occ. at T28 (%)	43.4	39.1	40.1	37.6	35.6	39.6	38.6

Table S8. Point symbol, boron occupancy and average ∠TOT of calcined EMM-59.

**T-site**	**Point Symbol**	**B Occ.**	**Avg.∠TOT** (°)
Si1	[$4.5^3.6.10$]	0	150.60
Si2	[$5^5.6$]	0	157.40
Si3	[$5^4.6^2$]	0	152.32
Si4	[$5^3.6^2.10$]	0	152.30
Si5	[$4.5^3.6.10$]	0	148.59
Si6	[$4.5^3.6.8$]	0	150.49
Si7	[$5^5.10$]	0	155.26
Si8	[$5^5.10$]	0	154.06
Si9	[$5^5.10$]	0	157.66
Si10	[$5^2.6^3.10$]	0	148.89
Si11	[$5^2.6^2.12^2$]	0	150.12
Si12	[$5^5.6$]	0	157.70
Si13	[$4.5^4.10$]	0	150.28
Si14	[$5^4.6.10$]	0	151.24
Si15	[$4.5^3.6^2$]	0.04	154.35
Si16	[$4.5^4.6$]	0	156.08
Si17	[$5^3.6^2.12$]	0	148.64
Si18	[$4.5^4.6$]	0	154.87
Si19	[$4.5^3.6.10$]	0	153.86
Si20	[$5^5.10$]	0	150.61
Si21	[$4.5^4.12$]	0.07	147.20
Si22	[$4.5^3.6.12$]	0	147.76
Si23	[$4^2.5^2.6.10$]	0	148.82
Si24	[$4^2.5^3.6$]	0	149.11
Si25	[$4^2.5^3.10$]	0.09	145.25
Si26	[$4^2.5^3.10$]	0.05	148.61
Si27	[$4^3.5^2.8$]	0	143.79
Si28	[$4^3.5^2.12$]	0.38	143.42

Table S9. Point symbol, boron occupancy and average ∠TOT of as-made SSZ-53 (**SFH**).[3]

T-site	Point Symbol	B Occ.	Avg. ∠TOT (°)
Si1	[$4.5^2.6^3$]	0	151.82
Si2	[$4^2.5^2.6.14$]	0.150	137.92
Si3	[$5^3.6^3$]	0	151.05
Si4	[$4^2.5^2.6.14$]	0.114	140.15
Si5	[$4.5^2.6^3$]	0	152.11
Si6	[$5^3.6^3$]	0	146.75
Si7	[$5^2.6^3.14$]	0	146.19
Si8	[$5^2.6^4$]	0	145.32

Table S10. Point symbol, boron occupancy and average ∠TOT of as-made SSZ-55 (**ATS**).[3]

**T-site**	**Point Symbol**	**B Occ.**	**Avg.∠TOT** (°)
Si1	[$4^2.6^4$]	0.141	151.95
Si2	[$4^2.6^3.12$]	0	146.84
Si3	[$4^2.6^3.12$]	0	146.20

Table S11. Point symbol, boron occupancy and average ∠TOT of as-made SSZ-56 (**SFS**).[3]

**T-site**	**Point Symbol**	**B Occ.**	**Avg. ∠TOT** (°)
Si1	[$4^2.5^3.10$]	0	147.54
Si2	[$5^4.6^2$]	0	152.35
Si3	[$5^5.6$]	0	156.31
Si4	[$5^5.6$]	0	153.19
Si5	[$4.5^2.6.10.12$]	0.092	149.80
Si6	[$5^4.6.12$]	0	154.93
Si7	[$4.5^2.6.10.12$]	0	151.72
Si8	[$4^2.5^2.6.10$]	0.127	144.08
Si9	[$4^2.5^2.6.10$]	0	146.60
Si10	[$5^5.10$]	0	151.39
Si11	[$5^4.6.10$]	0	150.38
Si12	[$5^5.12$]	0	156.62
Si13	[$4^2.5^3.10$]	0	148.15
Si14	[$5^3.6^2.10$]	0	153.82

Table S12. Point symbol, boron occupancy and average ∠TOT of as-made SSZ-58 (**SFG**).[3]

**T-site**	**Point Symbol**	**B Occ.**	**Avg. ∠TOT** (°)
Si1	$[5^2.6^3.7]$	0	159.38
Si2	$[4.5^2.6^2.7]$	0.083	158.69
Si3	$[5^2.6^4]$	0	152.70
Si4	$[4^2.5.6^2.10]$	0	149.94
Si5	$[5^2.6^4]$	0	163.37
Si6	$[5^2.6^4]$	0	166.25
Si7	$[4.5^2.6^3]$	0.094	157.57
Si8	$[4^2.5.6^2.10]$	0.058	148.94
Si9	$[5^2.6^3.7]$	0	158.89
Si10	$[5^2.6^4]$	0	171.35
Si11	$[4^2.5.6^3]$	0	149.49
Si12	$[4^2.5.6^2.10]$	0	150.46

Table S13. Point symbol, boron occupancy and average ∠TOT of as-made SSZ-59 (**SFN**).[3]

T-site	Point Symbol	B Occ.	Avg. ∠TOT (°)
Si1	[$4.5^2.6^3$]	0	150.11
Si2	[$4.5^2.6^3$]	0.064	151.97
Si3	[$5^2.6^3.14$]	0	145.60
Si4	[$5^3.6^3$]	0	144.70
Si5	[$5^2.6^4$]	0	146.08
Si6	[$5^3.6^3$]	0	150.62
Si7	[$4^2.5^2.6.14$]	0.127	139.16
Si8	[$4^2.5^2.6.14$]	0	141.80

Table S14. Point symbol, boron occupancy and average ∠TOT of as-made SSZ-82 (**SEW**).[4]

T-site	Point Symbol	B Occ.	Avg. ∠TOT (°)
Si1	[$4.5^2.6^2.12$]	0	151.74
Si2	[$4.5^2.6^3$]	0	147.88
Si3	[$4^2.5.6^2.12$]	0	152.25
Si4	[$4^2.5.6^2.12$]	0	146.26
Si5	[$4^2.5^2.6.10$]	0.250	146.06
Si6	[$4^3.5.6.10$]	0.340	139.94
Si7	[$4^2.5^3.10$]	0	150.97
Si8	[$5^4.6^2$]	0	161.92
Si9	[$4^2.5.6^3$]	0	155.37
Si10	[5.6^5]	0	157.91
Si11	[$5.6^4.10$]	0	161.43

Table S15. Point symbol, boron occupancy and average ∠TOT of as-made SSZ-87 (**IFW**).[5]

T-site	Point Symbol	B Occ.	Avg. ∠TOT (°)
Si1	$[4^2.5^2.6.10]$	0	146.39
Si2	$[4^3.5.6.10]$	0.151	144.29
Si3	$[4^2.5^2.6.8]$	0	149.58
Si4	$[4.5^2.6^2.8]$	0	151.12
Si5	$[5^3.6^2.8]$	0	158.10
Si6	$[4^2.5^2.6.8]$	0.354	145.64
Si7	$[4.5^4.6]$	0	153.82
Si8	$[4.5^4.6]$	0	159.55
Si9	$[4.5^4.10]$	0	150.88
Si10	$[4.5^4.6]$	0	163.15

Table S16. Point symbol, boron occupancy and average ∠TOT of calcined EMM-26 (**EWS**).[6]

**T-site**	**Point Symbol**	**B Occ.**	**Avg. ∠TOT** (°)
Si1	[$5^3.6.10^2$]	0	151.07
Si2	[$5^3.6.12^2$]	0	147.63
Si3	[$5^5.10$]	0	153.52
Si4	[$4.5^3.6^2$]	0.077	143.58
Si5	[$4^3.5^2.10$]	0.351	138.70
Si6	[$4.5^2.6^3$]	0.145	142.88
Si7	[$5^5.6$]	0	150.28

References

1. Diederichs, K. & Karplus, P. A. Improved R-factors for diffraction data analysis in macromolecular crystallography. *Nat Struct Mol Biol* **4**, 269–275 (1997).

2. Karplus, P. A. & Diederichs, K. Linking Crystallographic Model and Data Quality. *Science* **336**, 1030–1033 (2012).

3. Smeets, S. *et al.* Locating Organic Guests in Inorganic Host Materials from X-ray Powder Diffraction Data. *J. Am. Chem. Soc.* **138**, 7099–7106 (2016).

4. Xie, D., McCusker, L. B. & Baerlocher, C. Structure of the Borosilicate Zeolite Catalyst SSZ-82 Solved Using 2D-XPD Charge Flipping. *J. Am. Chem. Soc.* **133**, 20604–20610 (2011).

5. Smeets, S. *et al.* SSZ-87: A Borosilicate Zeolite with Unusually Flexible 10-Ring Pore Openings. *J. Am. Chem. Soc.* **137**, 2015–2020 (2015).

6. Guo, P. *et al.* Accurate structure determination of a borosilicate zeolite EMM-26 with two-dimensional 10 × 10 ring channels using rotation electron diffraction. *Inorg. Chem. Front.* **3**, 1444–1448 (2016).

7. Phillips, M. W., Gibbs, G. V. & Ribbe, P. H. The Crystal Structure of Danburite: A Comparison with Anorthite, Albite, and Reedmergnerite. *American Mineralogist* **59**, 79–85 (1974).

8. Foit, F. F., Phillips, M. W. & Gibbs, G. V. A Refinement of the Crystal Structure of Datolite,CaBSiO4(OH). *American Mineralogist* **58**, 909–914 (1973).

9. Ghose, S., Wan, C. & Ulbrich, H. H. Structural Chemistry of Borosilicates. I. Garrelsite, NaBa3SizB7016(OH)4: a Silicoborate with the Pentaborate IB501219- Polyanion. *Acta Cryst.* **B32**, 824–832 (1976).

10. Miyawaki, R., Nakai, I. & Nagashima, K. Structure of Homilite, Ca2.00(Fe0.90Mn0.03)B2.00Si2.00O9.86(OH)0.14. *Acta Cryst.* **C41**, 13–15 (1985).

11. Callegari, A., Giuseppetti, G., Mazzi, F. & Tadini, C. The refinement of the crystal structure of stillwellite: RE[BSiO5]. *Neues Jahrbuch für Mineralogie, Monatshefte* 49–57 (1992).

12. Xie, D. *et al.* Optimized Synthesis and Structural Characterization of the Borosilicate MCM-70. *J. Phys. Chem. C* **113**, 9845–9850 (2009).
